# Does Ras Activate Raf and PI3K Allosterically?

**DOI:** 10.3389/fonc.2019.01231

**Published:** 2019-11-15

**Authors:** Ruth Nussinov, Chung-Jung Tsai, Hyunbum Jang

**Affiliations:** ^1^Cancer and Inflammation Program, Leidos Biomedical Research, Inc., Frederick National Laboratory for Cancer Research, National Cancer Institute at Frederick, Frederick, MD, United States; ^2^Department of Human Molecular Genetics and Biochemistry, Sackler School of Medicine, Tel Aviv University, Tel Aviv, Israel

**Keywords:** allosteric, allostery, B-Raf, KRas, K-Ras, NORE1A, BRAF

## Abstract

The mechanism through which oncogenic Ras activates its effectors is vastly important to resolve. If allostery is at play, then targeting allosteric pathways could help in quelling activation of MAPK (Raf/MEK/ERK) and PI3K (PI3K/Akt/mTOR) cell proliferation pathways. On the face of it, allosteric activation is reasonable: Ras binding perturbs the conformational ensembles of its effectors. Here, however, we suggest that at least for Raf, PI3K, and NORE1A (RASSF5), that is unlikely. Raf's long disordered linker dampens effective allosteric activation. Instead, we suggest that the high-affinity Ras–Raf binding relieves Raf's autoinhibition, shifting Raf's ensemble from the inactive to the nanocluster-mediated dimerized active state, as Ras also does for NORE1A. PI3K is recruited and allosterically activated by RTK (e.g., EGFR) at the membrane. Ras restrains PI3K's distribution and active site orientation. It stabilizes and facilitates PIP_2_ binding at the active site and increases the PI3K residence time at the membrane. Thus, RTKs allosterically activate PI3Kα; however, merging their action with Ras accomplishes full activation. Here we review their activation mechanisms in this light and draw attention to implications for their pharmacology.

## Introduction

*Is allostery driving Ras activation of its effectors*? The presumption that this is the case is easy to understand. Active Ras binds its effectors, and direct binding always perturbs the structures, initiating and promoting dynamic and at least some conformational changes (1–4). The relevant question is though—does Ras binding promote signals that propagate, through some allosteric pathways, *and lead to a functional change*? That is, do these signals prompt conformational and dynamic changes that affect the active site and are the dominant mechanism of effector activation? Even though not directly observed, the premise in the community has been that this is likely to be the case.

**Figure 1:**
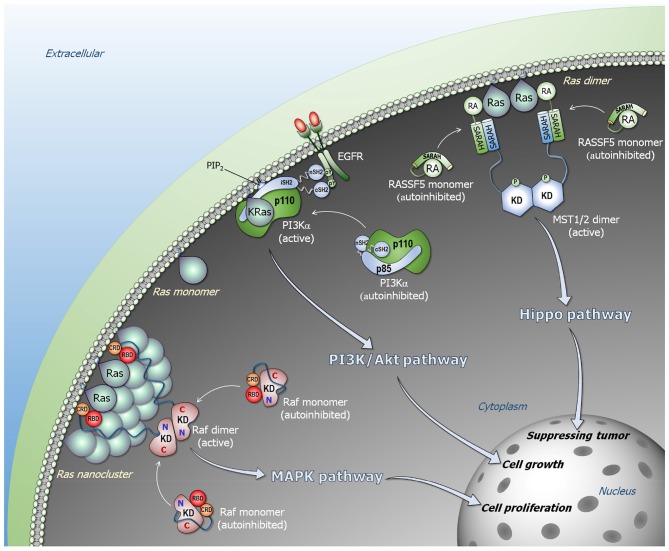
Ras signaling pathways. Ras forms nanoclusters and promotes Raf dimerization in the Raf/MEK/ERK (MAPK) pathway (lower left). Monomeric Raf is autoinhibited in cytosol, and the high-affinity Ras–RBD interaction releases the autoinhibition, activating Raf through side-by-side dimerization. PI3Kα is allosterically activated by EGFR (middle). The C-terminal phosphorylated tyrosine motif of EGFR liberates the SH2 domains of p85α regulatory subunit from the p110α catalytic subunit, releasing the autoinhibition of PI3Kα. Ras binding is not link to the allosteric activation of PI3Kα, but its binding contributes to further increase in the residence time of active PI3Kα at the membrane. NORE1A (RASSF5) is an adaptor protein and autoinhibited by its RA domain interacting with its SARAH domain (upper right). In the presence of proximal Ras molecules, the Ras–RA interaction liberates NORE1A SARAH to recruit MST1/2 SARAH, promoting MST1/2 dimerization through their kinase domains that activates MST1/2 via cross phosphorylation. In the presence of Hippo signal, the active MST1/2 kinase promotes phosphorylation cascade signal, leading to YAP1 phosphorylation and degradation that result in tumor suppressing.

This premise has recently been revisited. Experimental and computational data indicated that at least for phosphatidylinositide-3-kinase α (PI3Kα) this is *not* the case (5, 6). Indeed, PI3Kα is known to be recruited and activated by epidermal growth factor receptor (EGFR), a receptor tyrosine kinase (RTK), at the membrane (7, 8). For Raf the premise still prevails. Here we overview PI3Kα and Raf activation, as well as activation of Ras association domain family 5 (RASSF5, a.k.a. NORE1A) tumor suppressor (Figure 1). We suggest that these Ras effectors are not activated via allosteric activation through Ras interaction. Further, even though to date there are no data relating to other Ras effectors, we suspect that this holds. In the case of Raf, a long disordered linker joins the kinase domain with the regulatory domain containing the Ras binding domain (RBD) and the cysteine-rich domain (CRD), which attaches Raf to the membrane (9–11). Protein disorder inherently implies no preferred interactions, no matter the sequence length. In the absence of specific interactions between the linker and RBD and the kinase domain, no allosteric propagation can take place. If no allosteric propagation, it is like there is no linkage between the two domains. The high-affinity Ras–RBD interaction (12, 13)—vs. the low affinity autoinhibition—argues in favor of activation via a shift in Raf's population toward the Ras-bound active state. In the case of PI3Kα, it is allosterically activated by the binding of the phosphorylated EGFR C-terminal motif to PI3Kα's Src homology 2 (SH2) domains (7, 14, 15); not by Ras. These binding events promote a conformational change which relieves PI3Kα autoinhibition and recruit PI3Kα to the membrane. Notably, EGFR activates PI3Kα even in the absence of Ras (16), albeit to a lesser extent. Activation of NORE1A tumor suppressor resembles the activation of the Raf proteins (17, 18). Taken together, these lead us to suggest some guidelines as to when allostery may not be involved in activation in binding events. This is important, since the mechanisms of activation are considered in drug discovery (19–26). If allostery is at play, disrupting propagation pathways is often deliberated.

Below, we first provide a brief background of allosteric activation. Next, we discuss activation of three Ras effectors, Raf, PI3Kα and NORE1A, and why allostery is unlikely to be involved. Finally, we lay out guidelines relating to when allostery is unlikely.

## Allosteric Activation: Definition and Background

Classically, allosteric activation is defined as inducing a conformational change in the active site of the enzyme by binding at a location other than the active site. We suggested that if a conformational change is not observed, then it is likely due to limitations in the experimental approach used to detect a conformational change (27). Thus, with this definition, if Ras only has a role in recruiting the enzyme to the membrane, it would not be allostery since it does not elicit a conformation that alters the active site. Similarly, if Ras were to only restrict the orientation of the active site relative to the membrane to make productive catalysis more likely, by definition, this would also not be allostery because it would not involve a conformational change.

**Figure 2:**
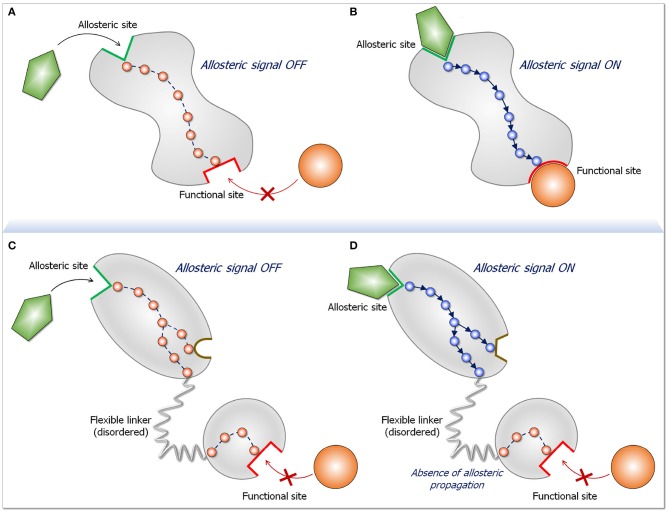
Schematic diagram for an allosteric propagation pathway and its absence in long disordered linkers. The top two panels display a two-state dynamic allosteric switch. Both states pre-exist in the population. In the absence of the ligand **(A)** the protein populates a conformation in the ligand-free state. Upon ligand binding at the allosteric site **(B)**, a functional switch that is in favor of a ligand-bound state initiates at the binding site and propagates down to the functional site. The two bottom panels **(C,D)** depict what happens when two domains are joined by a long, disordered linker. The two-state switch takes place only in the domain to which the allosteric ligand binds, but do not propagate down the linker. The reason for the absence of allosteric propagation through the long linker is that the disordered state is distributed in multiple conformations. Since in the disordered state there are no specific stabilized interactions, there is no preferred propagation pathway. Preferred propagation pathways are required for population shift. In practice, identification of an allosteric propagation pathway in the structure can be achieved through superposition of the two (active and inactive) structures and locating changes in interactions of residues along pathways extending from the allosteric site to the functional site.

Allostery is linked to structural perturbation events (27–37). The events can be covalent changes, such as mutations, allosteric post-translational modifications (PTMs) or covalent allosteric drugs (38–42), or non-covalent, such as binding of small molecules (drugs, membrane signaling lipids, cofactors, water molecules, ions) or macromolecules, such as proteins (43–45). Allosteric events can take place near or away from the functional (active, protein-protein interaction, etc.) site; both can elicit efficient communication and productive allosteric events (29, 46, 47). Whether covalent or non-covalent, the perturbation breaks and forms new atomic interactions. In turn, the local changes promote additional adjustments in the interactions in their environments. These remodeling perturbations propagate along multiple pathways, with favored paths extending to the functional site, shifting the ensemble, thereby accomplishing distinct conformational and dynamic changes that switch the protein from the inactive to the active state (*vice versa* for repressors) (Figure 2). Thus, conformational dynamics is implicitly at play since allosteric events take place by a shift of the ensemble from energetically less favored states to more favored ones. Notably, the active conformation already exists in the ensemble; however, the shifts in the ensemble that allostery promotes increase its population. This conformation is primed to bind the substrate.

Allostery involves propagation which argues that the location of the allosteric event with respect to the active site is an important factor in determining its efficiency. Even though compact structures can act as efficient vehicles in allosteric transmission, dynamic segments, such as loops, linkers and hinges, respond and can efficiently mediate function (48, 49). Ras effectors are multidomain proteins, and to date no statistics have been published of the distributions of cancer driver mutations in multidomain proteins with respect to the functional (active) site. We expect that driver mutations tend to occur in the domain whose function is targeted. Mutations occurring in the catalytic domain make the active site conformation substrate-favored; those in a regulatory domain that acts in autoinhibition through its interaction with the catalytic domain, would relieve the autoinhibition. We are unaware of driver mutations occurring in non-catalytic domains whose actions propagate via disordered linkers to alter the active site conformations, as would be the case if Ras binding to the Raf's RBD were to allosterically activate it. To our knowledge, to date no driver mutations have been identified in Raf's RBD to substitute for its interaction with Ras.

To explain how Ras activates Raf, we consider two fundamental physical tenets. First, every biomacromolecule exists in an ensemble of conformations. For rigid molecules the ensemble is more restricted; for flexible (especially disordered) it is broad. Second, the most stable state is the most populated state. The ensemble of Raf monomers can be classified into three states: an active Ras-bound “open” state; a free “open” conformational state, and an autoinhibited “closed” state, where the kinase domain is blocked by another segment of Raf which prohibits it from dimerization (Figure 1). In the absence of Ras, Raf largely populates the microensemble of the autoinhibited state; however, a certain fraction of the population will be in the free state. The autoinhibited state is unlikely to be stable, since if it were, it should be possible to experimentally determine it (by crystallization, NMR). This is not the case for the very stable Ras–RBD complex. In the presence of Ras, Raf is most highly populated in the Ras-bound state due to a shift of the free state fraction. The equilibrium between the autoinhibited state and the free state will then be restored by a certain shift of the autoinhibited state to the free state. Kinase domain dimerization can take place even in the absence of Ras; however, GTP-bound active Ras raises the otherwise low population of the active species, with the exposed kinase domain prepped for dimerization. Ras' action in NORE1A's activation resembles its action in Raf's activation (Figure 1).

Allostery is unlikely to be at play in Ras' contribution to PI3Kα activation either. RTK binds PI3Kα (Figure 1). Binding promotes relief of PI3Kα's autoinhibition and exposure of the active site to the lipid substrate at the membrane through conformational change (6). However, no conformational change in PI3Kα is stimulated by Ras. Consequently, it is reasonable to conclude that the mechanism of Ras' activation of PI3Kα is not allosteric. Thus, even though the mechanisms of Ras activation of its effectors differ, in none of those explored here allostery is incurred by Ras action. Below we provide the mechanistic details.

## Activation of Ras Effectors Raf, PI3K and NORE1A

### If Not Allostery, What Is Ras Role in PI3Kα Activation?

PI3Kα is a lipid kinase that phosphorylates phosphatidylinositol 4,5-bisphosphate (PIP_2_) to phosphatidylinositol 3,4,5-trisphosphate (PIP_3_). Binding of Akt protein kinase to PIP_3_ at the membrane is a key step in the AkT/mTOR signaling pathway leading to cell growth and proliferation. Inactive PI3Kα is a stable heterodimer. It consists of the p85α regulatory subunit and p110α catalytic subunit (6, 50) whose active site is blocked by p85α (15). Conformational changes, elicited primarily by the nSH2 domain of p85α, are a key step in PI3Kα activation (51, 52). These are the outcome of allosteric perturbation by EGFR (or another RTK). The phosphorylated tyrosine motif (pYxxM) in the C-terminal of RTK, interacts with high affinity with the nSH2 domain (7, 14). This interaction breaks the nSH2–p110α helical interface eliciting a conformational change that releases the nSH2 from p110α, as well as the p85α iSH2 domain from the p110α C2 domain, and the movement of the p110α's adaptor binding domain (ABD). iSH2 forms strong hydrophobic interactions and salt bridges with p110α's ABD, C2 and the kinase domains. Its rotation breaks its interaction with p110α's ABD consistent with hydrogen deuterium exchange mass spectrometry (HDX-MS) data (53). These conformational changes expose the PI3Kα membrane binding surface (5, 53–55). The mechanism of PI3Kα activation that we determined underscores the action of the RTK motif via its interaction with the nSH2 and the associated large conformational change. The release of nSH2 permits the C-lobe of the kinase domain to get away from the C2 domain, priming PI3Kα for phosphorylation of the PIP_2_ lipid substrate to PIP_3_ (15, 56). In oncogenic Ras, in the absence of RTK, calmodulin (CaM)'s phosphorylated tyrosine can similarly target the nSH2 (and cSH2 domains), recruiting and activating PI3Kα (57–59). Alternatively, EGFR overexpression can take place.

What is then Ras' role in PI3Kα activation? The RTK motif already accomplishes recruitment to the membrane with the coupled conformational change that relieves the autoinhibition and switches it from the inactive to the active state. The conformational change created by Ras binding is insignificant, and unlikely to play a role in activation. However, the PI3Kα population which is favorably positioned and oriented, primed for substrate binding and catalysis, is limited. We conclude that Ras binding serves to further increase the PI3K residence time at the membrane, stabilizing and facilitating PIP_2_ binding at the active site. Thus, RTKs allosterically activate PI3Kα; however, merging their action with Ras accomplishes full activation (5).

### If Not Allostery, How Does Ras Activate Raf?

Raf is a multidomain protein. It has a variable length N-terminal tail that was proposed to mediate calcium-dependent B-Raf homo- and hetero-dimerization (60), interact with the C-terminal (61), and be responsible for A-Raf low basal activity. It also includes the RBD and CRD domain that latches Raf to the membrane, a variable-length linker containing the Ser/Thr-rich segment (10, 11), and the kinase domain. In the inactive state, monomeric Raf is autoinhibited. It's likely autoinhibited organization has recently been reviewed (9) along with the supporting experimental data and theoretical considerations (11, 61–87).

The high affinity (nanomolar range) active Ras–Raf's RBD binding recruits Raf to the plasma membrane (61, 88). CRD's anchorage to the membrane (89–91) is stabilized by its ‘membrane insertion’ loop residues (89, 92) in an organization that is similar to the one it adopts when alone, not in the Ras–RBD context (89). The Raf-1 linker connecting RBD and CRD consists of only 6 residues that further constrain and stabilize the Ras–RDB-CRD organization at the membrane. No interactions are observed between KRas4B, including the farnesyl, and CRD. This is not the case for the HRas farnesyl group. However, different than KRas, HRas has also two palmitoyls, and the two membrane-anchored palmitoyls lend stability to the system (93). Additional interaction details of the different Ras–Raf systems have also been uncovered (59, 89, 94–96). In a favored orientation, KRas4B attaches to the membrane through its farnesylated hypervariable region (HVR) in a way such that the effector binding site faces away from the membrane and is largely exposed. This permits the RBD to interact at the effector binding site while the CRD is anchored at the membrane through its loop. The nanomolar affinity of the Ras–RBD interaction has been measured in solution. However, under physiological conditions at the membrane, fluctuations that take place and molecular dynamics (MD) simulations indicate that these can be significant. The tethered Ras–RBD-CRD organization reduces the Ras–RBD fluctuations, thus increases the residence times of the productive organization. The enhanced affinity promotes a population shift of the Raf ensemble toward this Ras-bound state, relieving the autoinhibition.

High affinity is not the sole factor controlling the relief of Raf's autoinhibition and population shift toward the open state. Whereas, the disordered linker (~180 residues in B-Raf; ~170 residues in Raf-1) between CRD and the kinase domain deters allosteric transmission, it also encodes residues whose phosphorylation enhances or abrogates the autoinhibition. Ser446 phosphorylation of B-Raf weakens the autoinhibition; phosphorylated Ser259 of Raf-1 is recognized by 14-3-3 proteins (86, 87, 97, 98), promoting the autoinhibition. Dephosphorylation by protein phosphatase 2A (PP2A) and protein phosphatase 1 (PP1) releases it, shifting the equilibrium toward open state (11, 80, 99–101). 14-3-3 also binds phosphorylated Ser621 of Raf-1 (86, 97, 98). The interaction of the N-terminal with the kinase domain is likely to be weak (9). Simultaneous binding at both sites can promote the autoinhibited state by stabilizing the interaction of the N-terminal segment and the kinase domain (11, 73, 87, 102–104). However, these distinct sites that assist in regulating the switch controlling the On/Off open/closed states, may not need such long linkers.

Taken together, this raises the question of *why long linkers*? We believe that the long linkers permit distancing the kinase domains from Ras–RBD-CRD at the membrane. The membrane is crowded. The linker efficiently connects the protein assemblies at the cytoplasm with signals communicated through receptor proteins, such as RTKs. In the cytoplasm, dimers of Raf kinase domains gather in large complexes, including mitogen-activated protein kinase (MEK) and extracellular signal-regulated kinase (ERK) dimers. Large scaffolding and adaptor proteins are also involved, e.g., kinase suppressor of Ras (KSR) (105, 106), IQ motif-containing GTPase activating protein (IQGAP) (107), heat shock protein (HSP90) (108), and galectin (109). All are large multidomain proteins that interact with additional proteins, such as IQGAP1 with Arp2/3 which stimulates branching of actin assemblies (110). The long linker provides an effective and pragmatic solution, enabling formation of clusters in the cytoplasm thus signaling efficiency. The large clusters are further favored by the water layer at the membrane surface which “pushes” or drives the proteins away from the membrane surface unless there are lipid-favoring residues at the protein surface, as in the case of CRD. The long linkers also vacate the requirement for Ras dimerization for Raf's activation. They allow Ras nanoclusters-mediated Raf's dimerization and activation (Figure 1).

Thus, rather than allostery, current data argues for a shift of the ensemble through release of the autoinhibited, closed state. In the absence of active Ras molecules, Raf mostly populates a closed autoinhibited state, with access to the kinase domain hindered by other segments. In the presence of Ras, the high affinity Ras–RBD interaction at the membrane shifts the ensemble. This mechanism is also supported by the dual 14-3-3 interaction, phosphorylation (dephosphorylation) experiments and mutational data [e.g., alanine and acidic substitutions at phosphorylation sites in the activation loop (73–75)]. It can explain why Raf evolved tight interaction with Ras and why Ras nanoclusters can function effectively in Raf's activation (111). It can also clarify how the large Raf assemblies with MAPK kinases and scaffolding proteins can form, act efficiently (112), and allow signaling dynamics (113) despite the crowded membrane surface.

### If Not Allostery, How Does Ras Activate NORE1A?

Different from Raf and PI3Kα, NORE1A (RASSF5) Ras effector is not a kinase, but essentially an adaptor protein, mediating the interactions of Ras and mammalian sterile 20-like kinase 1/2 (MST1/2). Ras-bound NORE1A activates the MST1/2 kinase (17, 114–118), which via the Hippo pathway phosphorylation cascade, leads to Yes-associated protein 1 (YAP1) phosphorylation and degradation. Overexpression of YAP1 induces cell proliferation (119). In the absence of active Ras, it is in a closed conformation, with its Ras association (RA) domain interacting weakly with the Sav-RASSF-Hippo (SARAH) domain. The linker between the two domains is short (5 residues) and contains a flexible hinge. In the presence of active Ras, the equilibrium shifts in favor of the tight Ras–RA interaction. The dissociated SARAH domain heterodimerizes with the MST1/2 SARAH domain. The tightly bound SARAH domain heterodimer releases the MST from its autoinhibited state, where the kinase domain interacts weakly with the MST SARAH domain. This shift in the MST ensemble from the inactive closed state to the open state permits kinase domain homodimerization and activation via trans-autophosphorylation. The affinity of the MST1/2 SARAH homodimer is lower than that of the hetero-SARAH dimer (120, 121), putting it under Ras control. NORE1A bridges Ras and MST (17), with Ras interaction acting to bring MST1/2 kinase domains into spatial proximity (18, 122), just like it activates Raf. Thus, rather than allostery activating NORE1A to promote its activation of MST kinase, the high (micromolar) affinity of the SARAH heterodimer drives the equilibrium toward NORE1A open active state, driving MST1/2 kinase activation via population shift.

## Concluding Remarks

Conformational ensembles and their shifts underlie biological processes (1–4, 30, 32, 123–131). Population shifts between two states due to differences in the stabilities follow the thermodynamic rule that systems are always driven to their free energy minima. In the case of the two Ras effectors described here, Raf and NORE1A, the higher stability of the interaction with Ras vs. that of the autoinhibited state drives the changes in the equilibrium. In the third, PI3K, Ras increases the population time at the membrane, facilitating PIP_2_ insertion. Understanding how Ras effectors are regulated is of paramount importance since it can help in pharmacological discovery. Ras has additional effectors, including Tiam1, RalGDS, AF6, RIN, and more. Scenarios involving high affinity to Ras and long disordered interdomain linkers are likely to discourage allosteric transmission. A tell-tale is the presence (or absence) of observable conformational changes (27, 132). If binding promotes a conformational change, allostery is likely at play (Figure 2). This is the case for RTK's phosphorylated motif promoting conformational change in the interactions of the nSH2 domain of the p85α, which expose p110α active site. On the other hand, in our MD simulations of PI3Kα RBD complexed with KRas4B, we observed only insignificant conformational changes in RBD making an allosteric mechanism unlikely, in line with experimental data discussed here.

Finally, Ras does not have an allosteric role for the three effectors discussed above. However, this is not necessarily always the case for Ras or Ras-like GTPases. One example is the Ras family GTPase RHEB that appears to have a primary role as an allosteric activator of the mTORC1 complex (133).

## Author Contributions

All authors listed have made a substantial, direct and intellectual contribution to the work, and approved it for publication.

### Conflict of Interest

The authors declare that the research was conducted in the absence of any commercial or financial relationships that could be construed as a potential conflict of interest.

## References

[1] Tsai CJ Nussinov R A unified view of “how allostery works”. PLoS Comput Biol. (2014) 10:e1003394 10.1371/journal.pcbi.1003394 24516370 PMC3916236

[2] Liu J Nussinov R. Allostery: an overview of its history, concepts, methods, and applications. PLoS Comput Biol. (2016) 12:e1004966. 10.1371/journal.pcbi.1004966 27253437 PMC4890769

[3] Nussinov R. Introduction to protein ensembles and allostery. Chem Rev. (2016) 116:6263–6. 10.1021/acs.chemrev.6b00283 27268255

[4] Boehr DD Nussinov R Wright PE. The role of dynamic conformational ensembles in biomolecular recognition. Nat Chem Biol. (2009) 5:789–96. 10.1038/nchembio.232 19841628 PMC2916928

[5] Buckles TC Ziemba BP Masson GR Williams RL Falke JJ. Single-molecule study reveals how receptor and ras synergistically activate PI3Kalpha and PIP3 signaling. Biophys J. (2017) 113:2396–405. 10.1016/j.bpj.2017.09.018 29211993 PMC5738497

[6] Zhang M Jang H Nussinov R. The structural basis for Ras activation of PI3Kalpha lipid kinase. Phys Chem Chem Phys. (2019) 21:12021–8. 10.1039/C9CP00101H 31135801 PMC6556208

[7] Nolte RT Eck MJ Schlessinger J Shoelson SE Harrison SC. Crystal structure of the PI 3-kinase p85 amino-terminal SH2 domain and its phosphopeptide complexes. Nat Struct Biol. (1996) 3:364–74. 10.1038/nsb0496-364 8599763

[8] Gabelli SB Echeverria I Alexander M Duong-Ly KC Chaves-Moreira D Brower ET. Activation of PI3Kalpha by physiological effectors and by oncogenic mutations: structural and dynamic effects. Biophys Rev. (2014) 6:89–95. 10.1007/s12551-013-0131-1 25309634 PMC4192660

[9] Nussinov R Zhang M Tsai CJ Liao TJ Fushman D Jang H. Autoinhibition in Ras effectors Raf, PI3Kalpha, and RASSF5: a comprehensive review underscoring the challenges in pharmacological intervention. Biophys Rev. (2018) 10:1263–82. 10.1007/s12551-018-0461-0 30269291 PMC6233353

[10] Terrell EM Morrison DK. Ras-mediated activation of the raf family kinases. Cold Spring Harb Perspect Med. (2019) 9:a033746. 10.1101/cshperspect.a033746 29358316 PMC6311149

[11] Lavoie H Therrien M. Regulation of RAF protein kinases in ERK signalling. Nat Rev Mol Cell Biol. (2015) 16:281–98. 10.1038/nrm3979 25907612

[12] Chuang E Barnard D Hettich L Zhang XF Avruch J Marshall MS. Critical binding and regulatory interactions between Ras and Raf occur through a small, stable N-terminal domain of Raf and specific Ras effector residues. Mol Cell Biol. (1994) 14:5318–25. 10.1128/MCB.14.8.5318 8035810 PMC359051

[13] Herrmann C Martin GA Wittinghofer A. Quantitative analysis of the complex between p21ras and the Ras-binding domain of the human Raf-1 protein kinase. J Biol Chem. (1995) 270:2901–5. 10.1074/jbc.270.7.2901 7852367

[14] Pauptit RA Dennis CA Derbyshire DJ Breeze AL Weston SA Rowsell S Murshudov GN. NMR trial models: experiences with the colicin immunity protein Im7 and the p85alpha C-terminal SH2-peptide complex. Acta Crystallogr D Biol Crystallogr. (2001) 57:1397–404. 10.1107/S0907444901012434 11567151

[15] Zhang M Jang H Nussinov R. The mechanism of PI3Kalpha activation at the atomic level. Chem Sci. (2019) 10:3671–80. 10.1039/C8SC04498H 30996962 PMC6430085

[16] Karasarides M Anand-Apte B Wolfman A A direct interaction between oncogenic Ha-Ras and phosphatidylinositol 3-kinase is not required for Ha-Ras-dependent transformation of epithelial cells. J Biol Chem. (2001) 276:39755–64. 10.1074/jbc.M102401200 11514541

[17] Liao TJ Tsai CJ Jang H Fushman D Nussinov R. RASSF5: an MST activator and tumor suppressor *in vivo* but opposite *in vitro*. Curr Opin Struct Biol. (2016) 41:217–24. 10.1016/j.sbi.2016.09.001 27643882

[18] Liao TJ Jang H Tsai CJ Fushman D Nussinov R. The dynamic mechanism of RASSF5 and MST kinase activation by Ras. Phys Chem Chem Phys. (2017) 19:6470–80. 10.1039/C6CP08596B 28197608 PMC5381522

[19] Stephens L Williams R Hawkins P. Phosphoinositide 3-kinases as drug targets in cancer. Curr Opin Pharmacol. (2005) 5:357–65. 10.1016/j.coph.2005.03.002 15963759

[20] Jambrina PG Bohuszewicz O Buchete NV Kolch W Rosta E. Molecular mechanisms of asymmetric RAF dimer activation. Biochem Soc Trans. (2014) 42:784–90. 10.1042/BST20140025 25109958

[21] Joseph EW Pratilas CA Poulikakos PI Tadi M Wang W Taylor BS. The RAF inhibitor PLX4032 inhibits ERK signaling and tumor cell proliferation in a V600E BRAF-selective manner. Proc Natl Acad Sci USA. (2010) 107:14903–8. 10.1073/pnas.1008990107 20668238 PMC2930420

[22] Poulikakos PI Zhang C Bollag G Shokat KM Rosen N. RAF inhibitors transactivate RAF dimers and ERK signalling in cells with wild-type BRAF. Nature. (2010) 464:427–30. 10.1038/nature08902 20179705 PMC3178447

[23] Vadas O Burke JE Zhang X Berndt A Williams RL. Structural basis for activation and inhibition of class I phosphoinositide 3-kinases. Sci Signal. (2011) 4:re2. 10.1126/scisignal.2002165 22009150

[24] Ruan Z Kannan N. Altered conformational landscape and dimerization dependency underpins the activation of EGFR by alphaC-beta4 loop insertion mutations. Proc Natl Acad Sci USA. (2018) 115:E8162–71. 10.1073/pnas.1803152115 30104348 PMC6126729

[25] Hubbard PA Moody CL Murali R. Allosteric modulation of Ras and the PI3K/AKT/mTOR pathway: emerging therapeutic opportunities. Front Physiol. (2014) 5:478. 10.3389/fphys.2014.00478 25566081 PMC4267178

[26] Tsai CJ Nussinov R. Allosteric activation of RAF in the MAPK signaling pathway. Curr Opin Struct Biol. (2018) 53:100–6. 10.1016/j.sbi.2018.07.007 30059805

[27] Nussinov R Tsai CJ. Allostery without a conformational change? Revisiting the paradigm. Curr Opin Struct Biol. (2015) 30:17–24. 10.1016/j.sbi.2014.11.005 25500675

[28] Guo J Zhou HX. Protein allostery and conformational dynamics. Chem Rev. (2016) 116:6503–15. 10.1021/acs.chemrev.5b00590 26876046 PMC5011433

[29] Smith IN Thacker S Seyfi M Cheng F Eng C. Conformational dynamics and allosteric regulation landscapes of germline PTEN mutations associated with autism compared to those associated with cancer. Am J Hum Genet. (2019) 104:861–78. 10.1016/j.ajhg.2019.03.009 31006514 PMC6506791

[30] Nussinov R Tsai CJ Ma B. The underappreciated role of allostery in the cellular network. Annu Rev Biophys. (2013) 42:169–89. 10.1146/annurev-biophys-083012-130257 23451894 PMC6407633

[31] Nussinov R Tsai CJ. Allostery in disease and in drug discovery. Cell. (2013) 153:293–305. 10.1016/j.cell.2013.03.034 23582321

[32] Del Sol A Tsai CJ Ma B Nussinov R. The origin of allosteric functional modulation: multiple pre-existing pathways. Structure. (2009) 17:1042–50. 10.1016/j.str.2009.06.008 19679084 PMC2749652

[33] Gardino AK Villali J Kivenson A Lei M Liu CF Steindel P. Transient non-native hydrogen bonds promote activation of a signaling protein. Cell. (2009) 139:1109–18. 10.1016/j.cell.2009.11.022 20005804 PMC2891250

[34] Astl L Verkhivker GM. Atomistic Modeling of the ABL kinase regulation by allosteric modulators using structural perturbation analysis and community-based network reconstruction of allosteric communications. J Chem Theory Comput. (2019) 15:3362–80. 10.1021/acs.jctc.9b00119 31017783

[35] Pfleger C Minges A Boehm M Mcclendon CL Torella R Gohlke H. Ensemble- and rigidity theory-based perturbation approach to analyze dynamic allostery. J Chem Theory Comput. (2017) 13:6343–57. 10.1021/acs.jctc.7b00529 29112408

[36] Ettayapuram Ramaprasad AS Uddin S Casas-Finet J Jacobs DJ. Decomposing dynamical couplings in mutated scFv antibody fragments into stabilizing and destabilizing effects. J Am Chem Soc. (2017) 139:17508–17. 10.1021/jacs.7b09268 29139290 PMC5998336

[37] Saleh N Saladino G Gervasio FL Clark T. Investigating allosteric effects on the functional dynamics of beta2-adrenergic ternary complexes with enhanced-sampling simulations. Chem Sci. (2017) 8:4019–26. 10.1039/C6SC04647A 30155211 PMC6094175

[38] Onel M Sumbul F Liu J Nussinov R Haliloglu T. Cullin neddylation may allosterically tune polyubiquitin chain length and topology. Biochem J. (2017) 474:781–95. 10.1042/BCJ20160748 28082425 PMC7900908

[39] Zhan C Qi R Wei G Guven-Maiorov E Nussinov R Ma B. Conformational dynamics of cancer-associated MyD88-TIR domain mutant L252P (L265P) allosterically tilts the landscape toward homo-dimerization. Protein Eng Des Sel. (2016) 29:347–54. 10.1093/protein/gzw033 27503954 PMC5001137

[40] Nussinov R Tsai CJ. The design of covalent allosteric drugs. Annu Rev Pharmacol Toxicol. (2015) 55:249–67. 10.1146/annurev-pharmtox-010814-124401 25149918

[41] Lu S Zhang J. Designed covalent allosteric modulators: an emerging paradigm in drug discovery. Drug Discov Today. (2017) 22:447–53. 10.1016/j.drudis.2016.11.013 27888140

[42] Bradshaw JM Mcfarland JM Paavilainen VO Bisconte A Tam D Phan VT. Prolonged and tunable residence time using reversible covalent kinase inhibitors. Nat Chem Biol. (2015) 11:525–31. 10.1038/nchembio.1817 26006010 PMC4472506

[43] Tsai CJ Nussinov R. Emerging allosteric mechanism of EGFR activation in physiological and pathological contexts. Biophys J. (2019) 117:5–13. 10.1016/j.bpj.2019.05.021 31202480 PMC6626828

[44] Zhao J Nussinov R Ma B. Antigen binding allosterically promotes Fc receptor recognition. MAbs. (2019) 11:58–74. 10.1080/19420862.2018.1522178 30212263 PMC6343797

[45] Liao TJ Jang H Fushman D Nussinov R. Allosteric KRas4B can modulate SOS1 Fast and slow ras activation cycles. Biophys J. (2018) 115:629–41. 10.1016/j.bpj.2018.07.016 30097175 PMC6103739

[46] Verkhivker GM. Biophysical simulations and structure-based modeling of residue interaction networks in the tumor suppressor proteins reveal functional role of cancer mutation hotspots in molecular communication. Biochim Biophys Acta Gen Subj. (2019) 1863:210–25. 10.1016/j.bbagen.2018.10.009 30339916

[47] Daily MD Upadhyaya TJ Gray JJ. Contact rearrangements form coupled networks from local motions in allosteric proteins. Proteins. (2008) 71:455–66. 10.1002/prot.21800 17957766 PMC5009369

[48] Papaleo E Saladino G Lambrughi M Lindorff-Larsen K Gervasio FL Nussinov R. The role of protein loops and linkers in conformational dynamics and allostery. Chem Rev. (2016) 116:6391–423. 10.1021/acs.chemrev.5b00623 26889708

[49] Ma B Tsai CJ Haliloglu T Nussinov R Dynamic allostery: linkers are not merely flexible. Structure. (2011) 19:907–17. 10.1016/j.str.2011.06.002 21742258 PMC6361528

[50] Thorpe LM Spangle JM Ohlson CE Cheng H Roberts TM Cantley LC Zhao JJ. PI3K-p110alpha mediates the oncogenic activity induced by loss of the novel tumor suppressor PI3K-p85alpha. Proc Natl Acad Sci USA. (2017) 114:7095–100. 10.1073/pnas.1704706114 28630349 PMC5502636

[51] Castellano E Downward J. RAS Interaction with PI3K: more than just another effector pathway. Genes Cancer. (2011) 2:261–74. 10.1177/1947601911408079 21779497 PMC3128635

[52] Murillo MM Zelenay S Nye E Castellano E Lassailly F Stamp G Downward J. RAS interaction with PI3K p110alpha is required for tumor-induced angiogenesis. J Clin Invest. (2014) 124:3601–11. 10.1172/JCI74134 25003191 PMC4109531

[53] Burke JE Perisic O Masson GR Vadas O Williams RL. Oncogenic mutations mimic and enhance dynamic events in the natural activation of phosphoinositide 3-kinase p110alpha (PIK3CA). Proc Natl Acad Sci USA. (2012) 109:15259–64. 10.1073/pnas.1205508109 22949682 PMC3458343

[54] Tsutsumi K Fujioka Y Tsuda M Kawaguchi H Ohba Y. Visualization of Ras-PI3K interaction in the endosome using BiFC. Cell Signal. (2009) 21:1672–9. 10.1016/j.cellsig.2009.07.004 19616621

[55] Wang J Yuan Y Zhou Y Guo L Zhang L Kuai X. Protein interaction data set highlighted with human Ras-MAPK/PI3K signaling pathways. J Proteome Res. (2008) 7:3879–89. 10.1021/pr8001645 18624398

[56] Jang H Banerjee A Chavan T Gaponenko V Nussinov R. Flexible-body motions of calmodulin and the farnesylated hypervariable region yield a high-affinity interaction enabling K-Ras4B membrane extraction. J Biol Chem. (2017) 292:12544–59. 10.1074/jbc.M117.785063 28623230 PMC5535030

[57] Nussinov R Tsai CJ Jang H. Oncogenic KRas mobility in the membrane and signaling response. Semin Cancer Biol. (2019) 54:109–13. 10.1016/j.semcancer.2018.02.009 29499269

[58] Chavan TS Jang H Khavrutskii L Abraham SJ Banerjee A Freed BC. High-Affinity Interaction of the K-Ras4B Hypervariable Region with the Ras Active Site. Biophys J. (2015) 109:2602–13. 10.1016/j.bpj.2015.09.034 26682817 PMC4699860

[59] Jang H Banerjee A Chavan TS Lu S Zhang J Gaponenko V. The higher level of complexity of K-Ras4B activation at the membrane. FASEB J. (2016) 30:1643–55. 10.1096/fj.15-279091 26718888 PMC4799498

[60] Terai K Matsuda M. The amino-terminal B-Raf-specific region mediates calcium-dependent homo- and hetero-dimerization of Raf. EMBO J. (2006) 25:3556–64. 10.1038/sj.emboj.7601241 16858395 PMC1538552

[61] Chong H Guan KL. Regulation of Raf through phosphorylation and N terminus-C terminus interaction. J Biol Chem. (2003) 278:36269–76. 10.1074/jbc.M212803200 12865432

[62] Bruder JT Heidecker G Rapp UR. Serum-, TPA-, and Ras-induced expression from Ap-1/Ets-driven promoters requires Raf-1 kinase. Genes Dev. (1992) 6:545–56. 10.1101/gad.6.4.545 1313769

[63] Fukui M Yamamoto T Kawai S Mitsunobu F Toyoshima K. Molecular cloning and characterization of an activated human c-raf-1 gene. Mol Cell Biol. (1987) 7:1776–81. 10.1128/MCB.7.5.1776 3299054 PMC365279

[64] Heidecker G Huleihel M Cleveland JL Kolch W Beck TW Lloyd P. Mutational activation of c-raf-1 and definition of the minimal transforming sequence. Mol Cell Biol. (1990) 10:2503–12. 10.1128/MCB.10.6.2503 2188091 PMC360607

[65] Ishikawa F Sakai R Ochiai M Takaku F Sugimura T Nagao M. Identification of a transforming activity suppressing sequence in the c-raf oncogene. Oncogene. (1988) 3:653–8. 2577866

[66] Ishikawa F Takaku F Hayashi K Nagao M Sugimura T. Activation of rat c-raf during transfection of hepatocellular carcinoma DNA. Proc Natl Acad Sci USA. (1986) 83:3209–12. 10.1073/pnas.83.10.3209 3010283 PMC323482

[67] Molders H Defesche J Muller D Bonner TI Rapp UR Muller R. Integration of transfected LTR sequences into the c-raf proto-oncogene: activation by promoter insertion. EMBO J. (1985) 4:693–8. 10.1002/j.1460-2075.1985.tb03685.x 4006904 PMC554244

[68] Schultz AM Copeland T Oroszlan S Rapp UR. Identification and characterization of c-raf phosphoproteins in transformed murine cells. Oncogene. (1988) 2:187–93. 3285297

[69] Stanton VP Jr Cooper GM. Activation of human raf transforming genes by deletion of normal amino-terminal coding sequences. Mol Cell Biol. (1987) 7:1171–9. 10.1128/MCB.7.3.1171 3561413 PMC365190

[70] Stanton VP Jr Nichols DW Laudano AP Cooper GM. Definition of the human raf amino-terminal regulatory region by deletion mutagenesis. Mol Cell Biol. (1989) 9:639–47. 10.1128/MCB.9.2.639 2710120 PMC362641

[71] Cutler RE Jr Stephens RM Saracino MR Morrison DK. Autoregulation of the Raf-1 serine/threonine kinase. Proc Natl Acad Sci USA. (1998) 95:9214–9. 10.1073/pnas.95.16.9214 9689060 PMC21318

[72] Tran NH Frost JA. Phosphorylation of Raf-1 by p21-activated kinase 1 and Src regulates Raf-1 autoinhibition. J Biol Chem. (2003) 278:11221–6. 10.1074/jbc.M210318200 12551923

[73] Tran NH Wu X Frost JA. B-Raf and Raf-1 are regulated by distinct autoregulatory mechanisms. J Biol Chem. (2005) 280:16244–53. 10.1074/jbc.M501185200 15710605

[74] Zhang BH Guan KL. Activation of B-Raf kinase requires phosphorylation of the conserved residues Thr598 and Ser601. EMBO J. (2000) 19:5429–39. 10.1093/emboj/19.20.5429 11032810 PMC314015

[75] Zhang BH Guan KL. Regulation of the Raf kinase by phosphorylation. Exp Lung Res. (2001) 27:269–95. 10.1080/019021401300054046 11293329

[76] Wan PT Garnett MJ Roe SM Lee S Niculescu-Duvaz D Good VM. Mechanism of activation of the RAF-ERK signaling pathway by oncogenic mutations of B-RAF. Cell. (2004) 116:855–67. 10.1016/S0092-8674(04)00215-6 15035987

[77] Kohler M Roring M Schorch B Heilmann K Stickel N Fiala GJ. Activation loop phosphorylation regulates B-Raf *in vivo* and transformation by B-Raf mutants. EMBO J. (2016) 35:143–61. 10.15252/embj.201592097 26657898 PMC4718462

[78] Mason CS Springer CJ Cooper RG Superti-Furga G Marshall CJ Marais R Serine and tyrosine phosphorylations cooperate in Raf-1, but not B-Raf activation. EMBO J. (1999) 18:2137–48. 10.1093/emboj/18.8.2137 10205168 PMC1171298

[79] Cook SJ Mccormick F. Inhibition by cAMP of Ras-dependent activation of Raf. Science. (1993) 262:1069–72. 10.1126/science.7694367 7694367

[80] Dhillon AS Meikle S Yazici Z Eulitz M Kolch W. Regulation of Raf-1 activation and signalling by dephosphorylation. EMBO J. (2002) 21:64–71. 10.1093/emboj/21.1.64 11782426 PMC125807

[81] Wu J Dent P Jelinek T Wolfman A Weber MJ Sturgill TW. Inhibition of the EGF-activated MAP kinase signaling pathway by adenosine 3',5'-monophosphate. Science. (1993) 262:1065–9. 10.1126/science.7694366 7694366

[82] Rommel C Clarke BA Zimmermann S Nunez L Rossman R Reid K. Differentiation stage-specific inhibition of the Raf-MEK-ERK pathway by Akt. Science. (1999) 286:1738–41. 10.1126/science.286.5445.1738 10576741

[83] Zimmermann S Moelling K. Phosphorylation and regulation of Raf by Akt (protein kinase B). Science. (1999) 286:1741–4. 10.1126/science.286.5445.1741 10576742

[84] Dhillon AS Pollock C Steen H Shaw PE Mischak H Kolch W. Cyclic AMP-dependent kinase regulates Raf-1 kinase mainly by phosphorylation of serine 259. Mol Cell Biol. (2002) 22:3237–46. 10.1128/MCB.22.10.3237-3246.2002 11971957 PMC133783

[85] Light Y Paterson H Marais R. 14–3-3 antagonizes Ras-mediated Raf-1 recruitment to the plasma membrane to maintain signaling fidelity. Mol Cell Biol. (2002) 22:4984–96. 10.1128/MCB.22.14.4984-4996.2002 12077328 PMC139778

[86] Michaud NR Fabian JR Mathes KD Morrison DK 14–3-3 is not essential for Raf-1 function: identification of Raf-1 proteins that are biologically activated in a 14–3-3- and Ras-independent manner. Mol Cell Biol. (1995) 15:3390–7. 10.1128/MCB.15.6.3390 7760835 PMC230573

[87] Tzivion G Luo Z Avruch J. A dimeric 14–3-3 protein is an essential cofactor for Raf kinase activity. Nature. (1998) 394:88–92. 10.1038/27938 9665134

[88] Herrmann C Horn G Spaargaren M Wittinghofer A. Differential interaction of the ras family GTP-binding proteins H-Ras, Rap1A, and R-Ras with the putative effector molecules Raf kinase and Ral-guanine nucleotide exchange factor. J Biol Chem. (1996) 271:6794–800. 10.1074/jbc.271.12.6794 8636102

[89] Li S Jang H Zhang J Nussinov R. Raf-1 Cysteine-rich domain increases the affinity of K-Ras/Raf at the membrane, promoting MAPK signaling. Structure. (2018) 26:513–25 e2. 10.1016/j.str.2018.01.011 29429878 PMC8183739

[90] Li ZL Prakash P Buck M. A “Tug of War” maintains a dynamic protein-membrane complex: molecular dynamics simulations of C-Raf RBD-CRD bound to K-Ras4B at an anionic membrane. ACS Cent Sci. (2018) 4:298–305. 10.1021/acscentsci.7b00593 29532030 PMC5832993

[91] Travers T Lopez CA Van QN Neale C Tonelli M Stephen AG Gnanakaran S. Molecular recognition of RAS/RAF complex at the membrane: role of RAF cysteine-rich domain. Sci Rep. (2018) 8:8461. 10.1038/s41598-018-26832-4 29855542 PMC5981303

[92] Improta-Brears T Ghosh S Bell RM. Mutational analysis of Raf-1 cysteine rich domain: requirement for a cluster of basic aminoacids for interaction with phosphatidylserine. Mol Cell Biochem. (1999) 198:171–8. 10.1023/A:1006981411691 10497893

[93] Thapar R Williams JG Campbell SL. NMR characterization of full-length farnesylated and non-farnesylated H-Ras and its implications for Raf activation. J Mol Biol. (2004) 343:1391–408. 10.1016/j.jmb.2004.08.106 15491620

[94] Li ZL Buck M. Computational modeling reveals that signaling lipids modulate the orientation of K-Ras4A at the membrane reflecting protein topology. Structure. (2017) 25:679–89 e2. 10.1016/j.str.2017.02.007 28286004 PMC6178820

[95] Abankwa D Gorfe AA Inder K Hancock JF. Ras membrane orientation and nanodomain localization generate isoform diversity. Proc Natl Acad Sci USA. (2010) 107:1130–5. 10.1073/pnas.0903907107 20080631 PMC2824305

[96] Gorfe AA Hanzal-Bayer M Abankwa D Hancock JF Mccammon JA. Structure and dynamics of the full-length lipid-modified H-Ras protein in a 1,2-dimyristoylglycero-3-phosphocholine bilayer. J Med Chem. (2007) 50:674–84. 10.1021/jm061053f 17263520

[97] Muslin AJ Tanner JW Allen PM Shaw AS. Interaction of 14–3-3 with signaling proteins is mediated by the recognition of phosphoserine. Cell. (1996) 84:889–97. 10.1016/S0092-8674(00)81067-3 8601312

[98] Rommel C Radziwill G Lovric J Noeldeke J Heinicke T Jones D. Activated Ras displaces 14–3-3 protein from the amino terminus of c-Raf-1. Oncogene. (1996) 12:609–19. 8637718

[99] Abraham D Podar K Pacher M Kubicek M Welzel N Hemmings BA. Raf-1-associated protein phosphatase 2A as a positive regulator of kinase activation. J Biol Chem. (2000) 275:22300–4. 10.1074/jbc.M003259200 10801873

[100] Jaumot M Hancock JF. Protein phosphatases 1 and 2A promote Raf-1 activation by regulating 14–3-3 interactions. Oncogene. (2001) 20:3949–58. 10.1038/sj.onc.1204526 11494123

[101] Ory S Zhou M Conrads TP Veenstra TD Morrison DK. Protein phosphatase 2A positively regulates Ras signaling by dephosphorylating KSR1 and Raf-1 on critical 14–3-3 binding sites. Curr Biol. (2003) 13:1356–64. 10.1016/S0960-9822(03)00535-9 12932319

[102] Matallanas D Birtwistle M Romano D Zebisch A Rauch J Von Kriegsheim A Kolch W. Raf family kinases: old dogs have learned new tricks. Genes Cancer. (2011) 2:232–60. 10.1177/1947601911407323 21779496 PMC3128629

[103] Dumaz N Marais R. Protein kinase A blocks Raf-1 activity by stimulating 14–3-3 binding and blocking Raf-1 interaction with Ras. J Biol Chem. (2003) 278:29819–23. 10.1074/jbc.C300182200 12801936

[104] Molzan M Ottmann C. Synergistic binding of the phosphorylated S233- and S259-binding sites of C-RAF to one 14–3-3zeta dimer. J Mol Biol. (2012) 423:486–95. 10.1016/j.jmb.2012.08.009 22922483

[105] MEK Binding to KSR promotes allosteric activation of BRAF. Cancer Discov. (2018) 8:385 10.1158/2159-8290.CD-RW2018-033 29475885

[106] Lavoie H Sahmi M Maisonneuve P Marullo SA Thevakumaran N Jin T. MEK drives BRAF activation through allosteric control of KSR proteins. Nature. (2018) 554:549–53. 10.1038/nature25478 29433126 PMC6433120

[107] Ren JG Li Z Sacks DB. IQGAP1 modulates activation of B-Raf. Proc Natl Acad Sci USA. (2007) 104:10465–9. 10.1073/pnas.0611308104 17563371 PMC1965536

[108] Stewart S Sundaram M Zhang Y Lee J Han M Guan KL. Kinase suppressor of Ras forms a multiprotein signaling complex and modulates MEK localization. Mol Cell Biol. (1999) 19:5523–34. 10.1128/MCB.19.8.5523 10409742 PMC84397

[109] Shalom-Feuerstein R Plowman SJ Rotblat B Ariotti N Tian T Hancock JF. K-ras nanoclustering is subverted by overexpression of the scaffold protein galectin-3. Cancer Res. (2008) 68:6608–16. 10.1158/0008-5472.CAN-08-1117 18701484 PMC2587079

[110] White CD Erdemir HH Sacks DB. IQGAP1 and its binding proteins control diverse biological functions. Cell Signal. (2012) 24:826–34. 10.1016/j.cellsig.2011.12.005 22182509 PMC3268868

[111] Nussinov R Tsai CJ Jang H. Is Nanoclustering essential for all oncogenic KRas pathways? Can it explain why wild-type KRas can inhibit its oncogenic variant? Semin Cancer Biol. (2019) 54:114–20. 10.1016/j.semcancer.2018.01.002 29307569

[112] Santos E Crespo P. The RAS-ERK pathway: a route for couples. Sci Signal. (2018) 11:eaav0917. 10.1126/scisignal.aav0917 30377222

[113] Nussinov R Jang H. Dynamic multiprotein assemblies shape the spatial structure of cell signaling. Prog Biophys Mol Biol. (2014) 116:158–64. 10.1016/j.pbiomolbio.2014.07.002 25046855 PMC4250281

[114] Avruch J Xavier R Bardeesy N Zhang XF Praskova M Zhou D. Rassf family of tumor suppressor polypeptides. J Biol Chem. (2009) 284:11001–5. 10.1074/jbc.R800073200 19091744 PMC2670104

[115] Avruch J Zhou D Fitamant J Bardeesy N Mou F Barrufet LR. Protein kinases of the Hippo pathway: regulation and substrates. Semin Cell Dev Biol. (2012) 23:770–84. 10.1016/j.semcdb.2012.07.002 22898666 PMC3489012

[116] Donninger H Schmidt ML Mezzanotte J Barnoud T Clark GJ. Ras signaling through RASSF proteins. Semin Cell Dev Biol. (2016) 58:86–95. 10.1016/j.semcdb.2016.06.007 27288568 PMC5034565

[117] Nussinov R Jang H Tsai CJ Liao TJ Li S Fushman D. Intrinsic protein disorder in oncogenic KRAS signaling. Cell Mol Life Sci. (2017) 74:3245–61. 10.1007/s00018-017-2564-3 28597297 PMC11107717

[118] Richter AM Pfeifer GP Dammann RH. The RASSF proteins in cancer; from epigenetic silencing to functional characterization. Biochim Biophys Acta. (2009) 1796:114–28. 10.1016/j.bbcan.2009.03.004 19344752

[119] Fallahi E O'driscoll NA Matallanas D. The MST/hippo pathway and cell death: a non-canonical affair. Genes. (2016) 7:E28. 10.3390/genes7060028 27322327 PMC4929427

[120] Huang CH Mandelker D Schmidt-Kittler O Samuels Y Velculescu VE Kinzler KW. The structure of a human p110alpha/p85alpha complex elucidates the effects of oncogenic PI3Kalpha mutations. Science. (2007) 318:1744–8. 10.1126/science.1150799 18079394

[121] Makbul C Constantinescu Aruxandei D Hofmann E Schwarz D Wolf E Herrmann C. Structural and thermodynamic characterization of Nore1-SARAH: a small, helical module important in signal transduction networks. Biochemistry. (2013) 52:1045–54. 10.1021/bi3014642 23331050

[122] Stieglitz B Bee C Schwarz D Yildiz O Moshnikova A Khokhlatchev A. Novel type of Ras effector interaction established between tumour suppressor NORE1A and Ras switch II. EMBO J. (2008) 27:1995–2005. 10.1038/emboj.2008.125 18596699 PMC2486280

[123] Kumar S Ma B Tsai CJ Sinha N Nussinov R. Folding and binding cascades: dynamic landscapes and population shifts. Protein Sci. (2000) 9:10–9. 10.1110/ps.9.1.10 10739242 PMC2144430

[124] Liu J Nussinov R. Energetic redistribution in allostery to execute protein function. Proc Natl Acad Sci USA. (2017) 114:7480–2. 10.1073/pnas.1709071114 28696318 PMC5530713

[125] Ma B Kumar S Tsai CJ Nussinov R. Folding funnels and binding mechanisms. Protein Eng. (1999) 12:713–20. 10.1093/protein/12.9.713 10506280

[126] Nussinov R Tsai CJ Csermely P. Allo-network drugs: harnessing allostery in cellular networks. Trends Pharmacol Sci. (2011) 32:686–93. 10.1016/j.tips.2011.08.004 21925743 PMC7380718

[127] Nussinov R Wolynes PG. A second molecular biology revolution? The energy landscapes of biomolecular function. Phys Chem Chem Phys. (2014) 16:6321–2. 10.1039/c4cp90027h 24608340

[128] Tsai CJ Del Sol A Nussinov R. Protein allostery, signal transmission and dynamics: a classification scheme of allosteric mechanisms. Mol Biosyst. (2009) 5:207–16. 10.1039/b819720b 19225609 PMC2898650

[129] Tsai CJ Kumar S Ma B Nussinov R. Folding funnels, binding funnels, and protein function. Protein Sci. (1999) 8:1181–90. 10.1110/ps.8.6.1181 10386868 PMC2144348

[130] Tsai CJ Ma B Nussinov R. Folding and binding cascades: shifts in energy landscapes. Proc Natl Acad Sci USA. (1999) 96:9970–2. 10.1073/pnas.96.18.9970 10468538 PMC33715

[131] Wei G Xi W Nussinov R Ma B. Protein ensembles: how does nature harness thermodynamic fluctuations for life? the diverse functional roles of conformational ensembles in the cell. Chem Rev. (2016) 116:6516–51. 10.1021/acs.chemrev.5b00562 26807783 PMC6407618

[132] Tsai CJ Del Sol A Nussinov R Allostery: absence of a change in shape does not imply that allostery is not at play. J Mol Biol. (2008) 378:1–11. 10.1016/j.jmb.2008.02.034 18353365 PMC2684958

[133] Yang H Jiang X Li B Yang HJ Miller M Yang A Dhar A Pavletich NP. Mechanisms of mTORC1 activation by RHEB and inhibition by PRAS40. Nature. (2017) 552:368–73. 10.1038/nature25023 29236692 PMC5750076

